# Induction of *Plasmodium falciparum*-Specific CD4^+^ T Cells and Memory B Cells in Gabonese Children Vaccinated with RTS,S/AS01_E_ and RTS,S/AS02_D_

**DOI:** 10.1371/journal.pone.0018559

**Published:** 2011-04-11

**Authors:** Selidji T. Agnandji, Rolf Fendel, Michaël Mestré, Michel Janssens, Johan Vekemans, Jana Held, Ferdinand Gnansounou, Sonja Haertle, Isabel von Glasenapp, Sunny Oyakhirome, Ludovic Mewono, Philippe Moris, Marc Lievens, Marie-Ange Demoitie, Patrice M. Dubois, Tonya Villafana, Erik Jongert, Aurelie Olivier, Joe Cohen, Meral Esen, Peter G. Kremsner, Bertrand Lell, Benjamin Mordmüller

**Affiliations:** 1 Medical Research Unit, Albert Schweitzer Hospital, Lambaréné, Gabon; 2 Institut für Tropenmedizin, Universität Tübingen, Tübingen, Germany; 3 GlaxoSmithKline Biologicals, Wavre, Belgium; 4 ImmunoVacc Consulting, Brussels, Belgium; 5 PATH Malaria Vaccine Initiative, Bethesda, Maryland, United States of America; The George Washington University Medical Center, United States of America

## Abstract

The recombinant circumsporozoite protein (CS) based vaccine, RTS,S, confers protection against *Plasmodium falciparum* infection in controlled challenge trials and in field studies. The RTS,S recombinant antigen has been formulated with two adjuvant systems, AS01 and AS02, which have both been shown to induce strong specific antibody responses and CD4 T cell responses in adults. As infants and young children are particularly susceptible to malaria infection and constitute the main target population for a malaria vaccine, we have evaluated the induction of adaptive immune responses in young children living in malaria endemic regions following vaccination with RTS,S/AS01_E_ and RTS,S/AS02_D_. Our data show that a CS-specific memory B cell response is induced one month after the second and third vaccine dose and that CS-specific antibodies and memory B cells persist up to 12 months after the last vaccine injection. Both formulations also induced low but significant amounts of CS-specific IL-2^+^ CD4^+^ T cells one month after the second and third vaccine dose, upon short-term *in vitro* stimulation of whole blood cells with peptides covering the entire CS derived sequence in RTS,S. These results provide evidence that both RTS,S/AS01_E_ and RTS,S/AS02_D_ induced adaptive immune responses including antibodies, circulating memory B cells and CD4^+^ T cells directed against *P. falciparum* CS protein.

**Trial Registration:**

ClinicalTrials.gov NCT00307021

## Introduction

Vaccination against *Plasmodium falciparum* (*P. falciparum*) infection and disease with an efficacious adjuvanted protein vaccine is an efficient and feasible strategy to reduce the burden of malaria in endemic regions. The candidate vaccine RTS,S targets the circumsporozoite protein (CS) in the pre-erythrocytic stage of the parasite and is based on a recombinant fusion protein (RTS) comprising the CS central tandem repeats and the C-terminal regions of CS fused to the N-terminal of hepatitis B surface antigen [1]. Co-expression of this fusion protein with native HepB surface antigen (S) results in the spontaneous formation of RTS,S virus-like particles. When formulated with the AS02 adjuvant, RTS,S was capable of inducing strong adaptive immune responses and protection against parasite challenge [2], [3]. The AS02 adjuvant is composed of an oil-in-water emulsion, the TLR4 ligand monophosporyl lipid A (MPL) and the saponin QS21 [1]. A number of clinical trials using the RTS,S/AS02 formulation have shown significant protection from infection in controlled challenge [3]–[6], or in field studies performed in adults and children living in malaria endemic regions [7]–[9]. New vaccine formulations were assessed for their ability to induce improved CS-specific immune responses. A second adjuvant named AS01 (which includes liposomes, MPL and QS21) was selected on the basis of its ability to induce comparable CS-specific antibody responses and greater T cell responses in small animal models and non human primates compared to the AS02 formulation [10], [11]. This was confirmed in a phase IIa controlled challenge study performed in healthy malaria naïve adults, where the RTS,S/AS01 formulation was shown to be safe and well tolerated, to induce strong humoral and cellular immune responses and to provide an improved protection against *P. falciparum* challenge as compared to RTS,S/AS02 [3]. Recent studies have also demonstrated that RTS,S adjuvanted with AS01 or AS02 is safe and highly immunogenic in adults [12] as well as in young children living in malaria endemic regions, and can reduce both infection rates and disease severity [13].

Both antibodies and T cell pre-erythrocytic responses have been shown to confer protection against *Plasmodium* infection in small animal studies [14]–[21]. The immune mechanisms underlying protection in humans have not been formally identified but recent evidence suggests that anti-CS mediated protection in adults depends on both strong antibodies and CD4^+^ T cell responses [3]. Since pediatric populations in endemic areas constitute the main target group for a malaria vaccine, it is important to investigate immune responses induced by RTS,S vaccines in this age group and also provide a better understanding of immune mechanisms which mediate protection. In this study we have investigated the impact of RTS,S vaccination on the induction of CS-specific antibodies, circulating memory B cells and CD4^+^ T cell responses in children aged 18 months to 4 years, vaccinated with AS01_E_ or AS02_D_ based formulations. While the rationale for investigating CD4^+^ T cell responses is based on their potential role in protection against infection in adults [3], [6], memory B cell responses and their relation to circulating antibody titers have not been evaluated with the RTS,S candidate vaccine.

## Materials and Methods

The supporting CONSORT checklist for this trial is available as supporting information; see Checklist S1. The protocol of this trial was posted with a previous publication [22].

### Ethics statement

The ethics committee of the International Foundation of the Albert Schweitzer Hospital of Lambaréné and the Western Institutional Review Board (USA) approved the study protocol. The trial was undertaken following the International Conference on Harmonisation of Good Clinical Practice guidelines. GSK Biologicals, Rixensart, Belgium, monitored the trial. In addition, a local safety monitor and a data and safety monitoring board closely reviewed the conduct and results of the trial.

### Study design and sampling

This trial has been described in detail in a previous publication [22]. It consisted of a phase II randomized, double blind study designed to document safety, reactogenicity and immunogenicity of the RTS,S/AS01_E_ and RTS,S/AS02_D_ formulations administered intramuscularly at 0, 1 and 2 months in children aged 18 months to 4 years in Lambaréné, Gabon. The primary endpoint of the trial was safety. The immunological analyses presented here were exploratory endpoints. A total of 180 eligible children were randomly assigned to a treatment group on the day of first vaccination. Because of limited volumes of blood, each group was randomly divided into two subgroups for evaluation of memory B cell responses using Enzyme Linked Immunospot Assay (ELISPOT) or T cell responses by detection of intracellular cytokine expression using whole blood. Blood samples were collected in lithium-heparin tubes before vaccination, one month post doses 2 and 3 and 12 month post dose 3 (study month 14). Peripheral blood mononuclear cells (PBMCs) for use in the memory B cells ELISPOT assay were isolated using Ficoll-Paque PLUS (GE healthcare, Germany).

### Detection of anti-CS and anti-HBs antibodies

Blood samples were collected before vaccination, one month post doses 2 and 3 and 12 months post dose 3. In all participants, serum antibodies to the NANP repeat region of CS (B cell epitope) were measured by a standard, validated enzyme-linked immunosorbent assay (ELISA) using plates adsorbed with the recombinant antigen R32LR that contains the sequence [NVDP(NANP)15]2LR, at a GSK validated laboratory (CEVAC, University of Ghent, Belgium). Titres were calculated using a reference standard curve with a 4 parameter logistic fitting algorithm and expressed in EU/ml, with cut-off for seropositivity set at 0.5 EU/ml [23]. Anti-hepatitis B surface antigen (HBs) antibody levels were measured with an in-house developed ELISA described previously [24].

### Detection of CS specific IgG secreting cells

The assay used for detection of circulating antigen specific B cells is based on a published method [25]. Briefly, fresh human PBMCs isolated by Ficoll density gradient were cultured at a concentration of 10^6^ cells/ml for 7 days in RPMI 1640 containing 1 mM sodium pyruvate, non-essential amino acids, 2 mM L-glutamine, 100 IU/ml penicillin, 100 µg/ml streptomycin, 50 µM 2-mercapto-ethanol and 10% Fetal Calf serum in the presence of 2.5 µg/ml CpG 2006 (TIB – MOLBIOL, Germany) and 10 ng/ml IL-15 (R&D Systems, Germany) at 37°C, 5% CO_2_ humidify atmosphere in 24 well culture plates. Cultured cells were then counted and transferred onto 96 well ELISA plates coated with 100 µl of 10 µg/ml R32LR or with 5 µg/ml of γ chain specific goat anti-human IgG (Sigma, Germany) for 3 hours at 37°C and saturated with PBS containing 3% BSA. CS-antigen specific immune responses were measured in triplicates over a period of three hours. A starting concentration of 10^6^ cells/well followed by two three-fold serial dilutions (333000, 111000 cells/well) was used for non-coated and antigen-coated wells (if available, but less in case of lower cell numbers extracted from the pediatric samples). For the IgG positive control, threefold serial dilutions were done with 27,000 cells/well as the highest density, followed by a series of three three-fold dilutions (9000, 3000, 1000 cells/well). Plates were washed and incubated with a biotin-labeled goat anti-human IgG (Sigma, Germany) followed by streptavidin-alkaline phosphatase (Roche, Germany). After a final washing step, wells were covered with a 0.6% agarose solution in 1 M 2-amino-2-methyl-1-propanol, 1.58 mM MgCl_2_, 0.01% Triton X-405 and 2.3 mM 5-Bromo-4-chloro-3-indolyl phosphate *p*-toluidine, pH 10.25. Plates were then incubated for 3 hours at 37°C for spot development, digitally photographed and counted manually using ImageJ software (National Institutes of Health, USA). The frequency of B lymphocytes expressing R32LR specific IgG was expressed as a number of antigen-specific cells per total IgG expressing cells after substraction of background responses. Samples which had less than 2000 IgG^+^ spots per million PBMCs were not considered for analysis.

### Whole blood Intracellular cytokine staining and flow cytometry

Intracellular cytokine staining (ICS) was used to assess cell-mediated immune responses, using a previously described method [26]. Just after collection, samples of 350 µl undiluted whole blood were incubated at 37°C with a pool of overlapping 15-mer peptides spanning the CS sequence in RTS,S (1.25 µg/ml per peptide), medium (negative control) or staphylococcal enterotoxin B (SEB, 0.25 µg/ml, positive control) for 2 hours in the presence of 1 µg/ml of anti-CD28 and anti-CD49d antibodies. Then, cytokine secretion inhibitor (Brefeldin A) was added at 1 µg/ml and incubated overnight. Following this, red blood cells were lysed and white blood cells were washed, fixed, and cryopreserved, and stored for cytometric analysis. Later, cells were thawed in bath, washed and permeabilised. The cells were then incubated with fluorescence-conjugated antibodies specific to CD3, CD4, CD8, CD40L, IFN-γ, IL-2 and TNF-α (BD biosciences ). After washing, flow cytometric acquisition was performed on a BD™ LSR II flow cytometer (BD biosciences, CA, USA), and analysis were completed with BD™ Diva software (BD biosciences, CA, USA) or FlowJo software (Tree Star™ Inc). Results are reported as the frequency of CD4^+^ and CD8^+^ T cells expressing at least CD40L, IFN- γ, IL-2 or TNF-α. A child was considered to be a responder if, upon CS antigen whole blood stimulation, the frequency of CS-specific cytokine expressing T cells was greater than or equal to the geometric mean + 3 standard deviations of cytokine expressing T cells in unstimulated samples.

### Statistical analysis

Immunogenicity analyses presented here were performed on the According To Protocol cohort for immunogenicity which included all subjects that received 3 doses of study vaccine according to protocol and for whom valid immunogenicity measurements were available.

The frequency of CS-specific B cells and T cells were summarized by descriptive statistics and tabulated by study group and timepoint. Between groups comparisons were performed by Wilcoxon Rank Sum tests (ie Mann-Whitney-U test, 2 sided, alpha 0.05). Within group comparisons were performed by Wilcoxon Signed Ranks test.

Spearman's rank correlation co-efficient (R) was used to assess the correlation between the log of anti-CS IgG level and log of the frequency of circulating memory B cells.

No adjustment for multiplicity of analysis was done.

## Results

**Figure 1:**
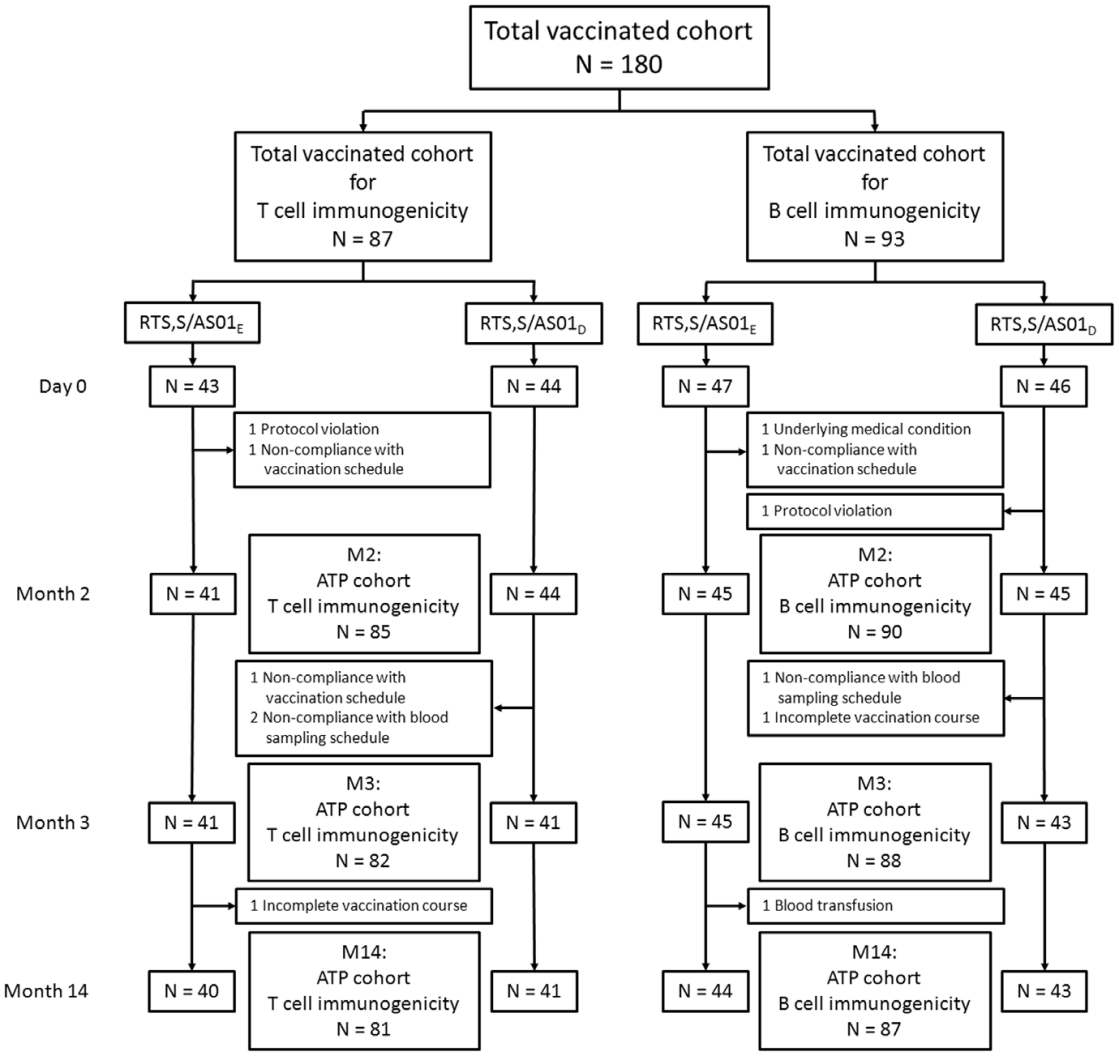
Flow of participants through the study.

Figure 1 shows the flow of subjects through the study.

### RTS,S vaccines induce circulating memory B cells producing CS-specific IgG

Vaccination with RTS,S has been shown to induce persistent antibody responses to CS in malaria exposed Gambian adults [27] and children in Mozambique [23]. The antibody responses to CS and HBs in children vaccinated with RTS,S/AS01_E_ and RTS,S/AS02_D_ in this study have been reported previously [22]. For information, all subjects from the B cell memory subset were anti-CS seropositive after the second vaccination with both vaccine formulations. In this group of children, the geometric mean anti-CS antibody concentration peaked after the third immunization for RTS,S/AS01_E_ and RTS,S/AS02_D_ (GMT 196 EU/ml 95% CI: 144–265 and 174 EU/ml 95% CI: 130–233 respectively) and all subjects remained seropositive for anti-CS antibodies one year after the last vaccine administration (GMT 15 EU/ml 95% CI: 10–24 and 19 EU/ml 95% CI: 12–31 respectively at month 14 of the study).

To investigate whether vaccination with RTS,S/AS01_E_ and RTS,S/AS02_D_ induced a memory B cell response in children, the presence of circulating memory B cells producing IgG specific for the *P. falciparum* CS protein was evaluated in this study using *in vitro* maturated memory B cells to detect antibody secreting cells by ELISPOT. Anti-CS antibody secreting cells were detected only in a few subjects, in low numbers, before the first vaccine administration (Table 1). At one month after the second RTS,S vaccination, memory B cells secreting anti-CS specific IgG were detected in the majority of study participants. ELISPOT responses to the CS antigen were significantly higher than pre-vaccination values after the second and third immunization in RTS,S/AS01_E_ and RTS,S/AS02_D_ vaccinated children. At one month post dose 2 the number of CS-specific circulating memory B cells was significantly higher in the RTS,S/AS01_E_ group compared to RTS,S/AS02_D_ group, but this difference was not seen at later timepoints. One year after last vaccination (month 14), the frequency of CS-specific circulating memory B cells had decreased in both groups but was still significantly higher than pre-vaccination values.

**Table 1:** Induction of memory B cell responses following vaccination with RTS,S/AS01_E_ and RTS,S/AS02_D_ (ATP cohort for immunogenicity - B cell subset).

	RTS,S/AS01_E_		RTS,S/AS02_D_		RTS,S/AS01_E_ vs RTS,S/AS02_D_ comparison
	N	Median (Q1–Q3)	N	Median (Q1–Q3)	p-value
**Pre**	17	0 (0–20)	23	0 (−49–0)	0.12
**Month 2**	31	1692 (345–4997)***	28	183 (0–1429)***	0.013
**Month 3**	29	1330 (466–2857)***	32	1795 (662–3204)***	0.38
**Month 14**	25	27 (0–2451)*	27	829 (25–1637)***	0.2

Footnote: Number of CS-specific spots per million total IgG-positive cells are presented.

*p<0.05.

***p<0.001, statistically significant differences against pre-vaccination values.

### Influence of prior vaccination with hepatitis B vaccine on the levels of circulating CS-specific memory B cells induced by RTS,S/AS01_E_ and RTS,S/AS02_D_

As, in this study, prior exposure to hepatitis B vaccination was associated with higher levels of CS-specific antibodies [22], it was investigated whether the hepatitis B vaccination status also had an impact on circulating CS-specific memory B cell responses in ELISPOT. Such an effect was not detected here: similar frequencies of CS-specific circulating memory B cells induced by RTS,S/AS01_E_ and RTS,S/AS02_D_ were observed in hepatitis B vaccinated and unvaccinated children. For example, at month 3 (one month post dose 3), the median (Q1–Q3) number of CS-specific memory B cells in children in the RTS,S/AS01E group previously vaccinated (N = 16) or not (N = 10) with hepatitis B vaccine was, respectively 1617 (594–3718) and 1496 (525–4657); in children in the RTS,S/AS02D group hepatitis B vaccinated (N = 16) or not (N = 16) was 1496 (525–4657) and 2071 (1075–3163), respectively.

### Assessment of the correlation between the frequency of CS-specific circulating memory B cells and CS-specific antibody titers

**Figure 2:**
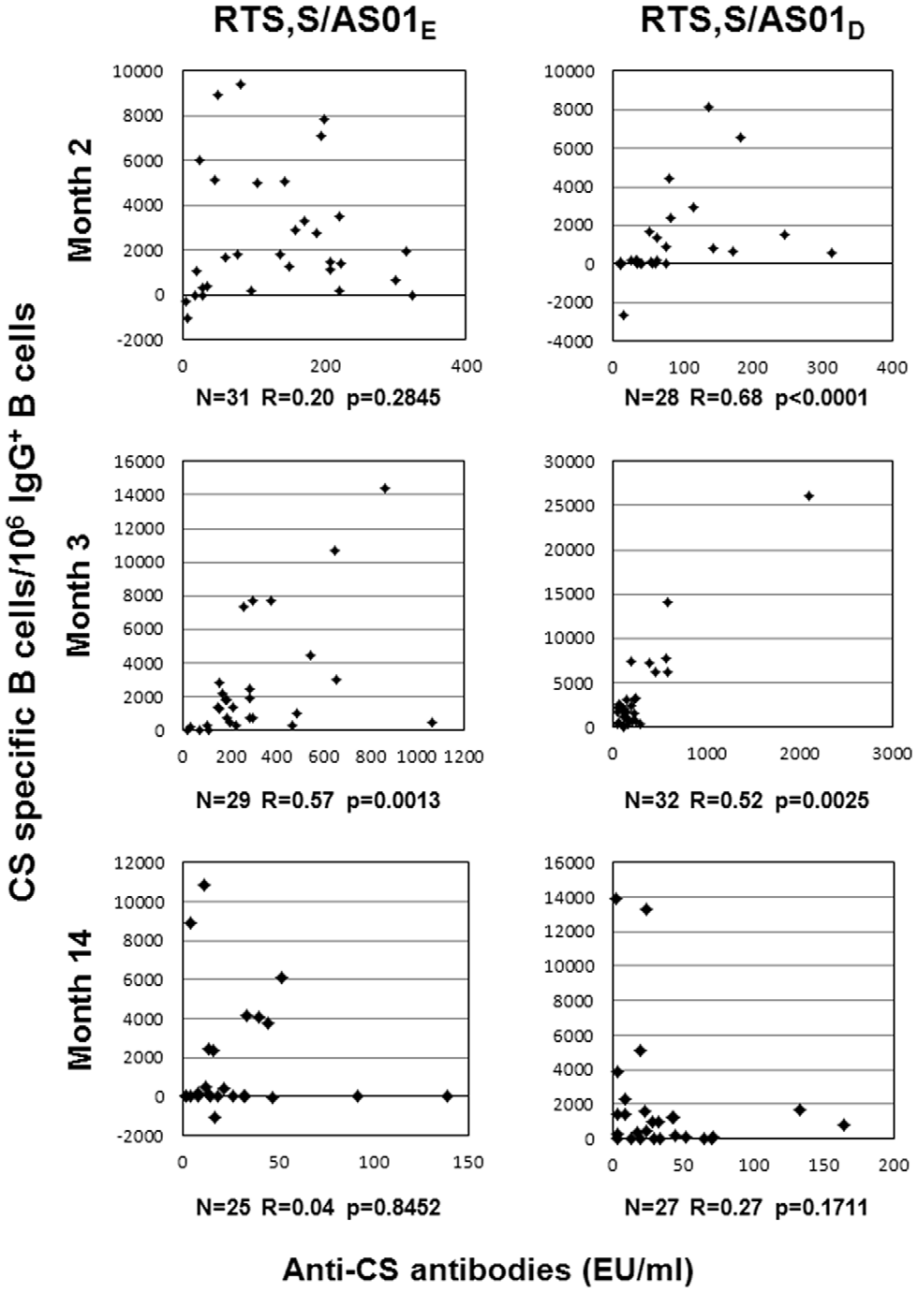
Association between CS-specific antibody responses and the frequency of CS-specific IgG^+^ memory B cells. The relationship between CS-specific antibody responses and the frequency of circulating CS-specific memory B cells was analysed at one month post dose 2 (month 2), one month post dose 3 (month 3) and one year post dose 3 (month 14). Data represent the CS-specific antibody level of each subject versus the corresponding frequency of CS-specific circulating memory B-cells for RTS,S/AS01_E_ and RTS,S AS02_D_ vaccine formulations. Number of responding subjects (n), Spearman correlation coefficient (R) and p-value are indicated.

Correlations between antigen-specific antibody levels and memory B cells have been reported in adults [28] and infants [29] for some vaccines. The relationship between CS-specific antibody responses and the frequency of circulating CS-specific memory B cells was investigated in this study (Figure 2). Analysis using Spearman's test (R) was done on subjects who were positive for CS-specific memory B cells. Statistically significant positive correlations between the CS-specific antibody level of each subject and the corresponding frequency of CS-specific circulating memory B cells were found at study month 2 (one month post dose 2) in the RTS,S/AS02D group only and at month 3 (one month post dose 3) in both study groups, but not at study month 14.

### RTS,S/AS01_E_ and RTS,S/AS02_D_ induce CS-specific CD4^+^ T cell responses

**Figure 3:**
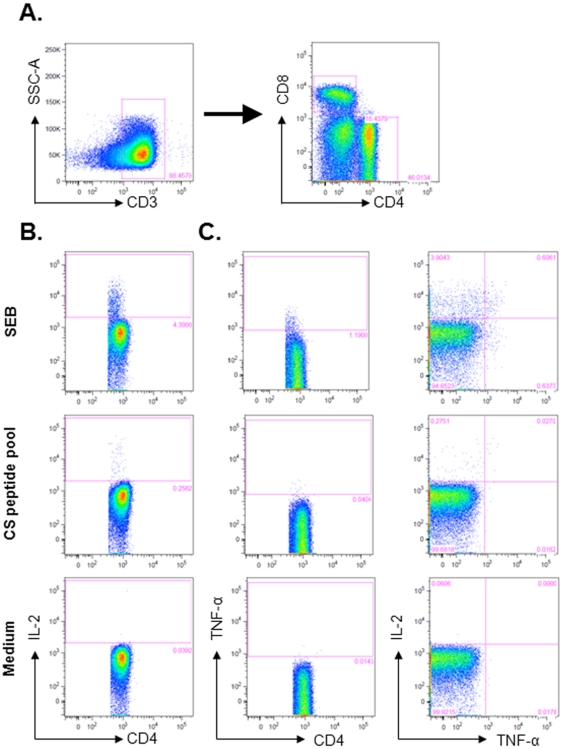
Whole-blood intracellular cytokine detection by flow cytometry. Whole-blood intracellular cytokine detection by flow cytometry was performed following overnight stimulation with SEB (positive control), the CS peptide pool or medium. (A) Cytokine production by CS-specific CD4^+^ T cells was determined by first dividing the CD3^+^ T cells in the lymphocyte gate into CD4^+^ or CD8^+^ T cells. (B) CD3^+^ CD4^+^ CD8^−^ T cells were analyzed with respect to the production of IL-2 and TNF-α. The unstimulated sample shows background levels of cytokine production, while stimulation with SEB shows strong production of IL-2 or TNF-α by CD4^+^ T cells. When restimulated with the CS peptide pool, the production of IL-2 by CD4^+^ T cells and low production of TNF-α was detected. (C) CD3^+^ CD4^+^ CD8^−^ T cells were analyzed for polyfunctional responses by simultaneous detection of IL-2 and TNF-α. While stimulation with SEB induces CD4^+^ T cells which produce the single cytokines and cells which produce both IL-2 and TNF-α, restimulation with the CS peptide pool induces mainly CD4^+^ T cells producing IL-2 only and a small fraction of cells is producing both IL-2 and TNF-α. The numbers in the quadrant gates of the plots denominates each distinct population based on their cytokine production. Samples from the same subject are shown, with responses at one month post dose 2. Results shown are representative of the range of responses seen with all subjects studied.

T cell responses to the CS antigen have been reported to be associated with protection in *P. falciparum* controlled challenge trials [3], [6]. Cellular responses to the CS antigen were therefore investigated in young children vaccinated with RTS,S formulated in AS01_E_ or AS02_D_ before vaccination and one month after the second and third vaccination. The detection of the intracellular cytokine expression following overnight stimulation with SEB (positive control), the CS peptide pool or medium (negative control) was performed by flow cytometry. Gating strategy and representative Scatterplots are presented in Figure 3.

**Figure 4:**
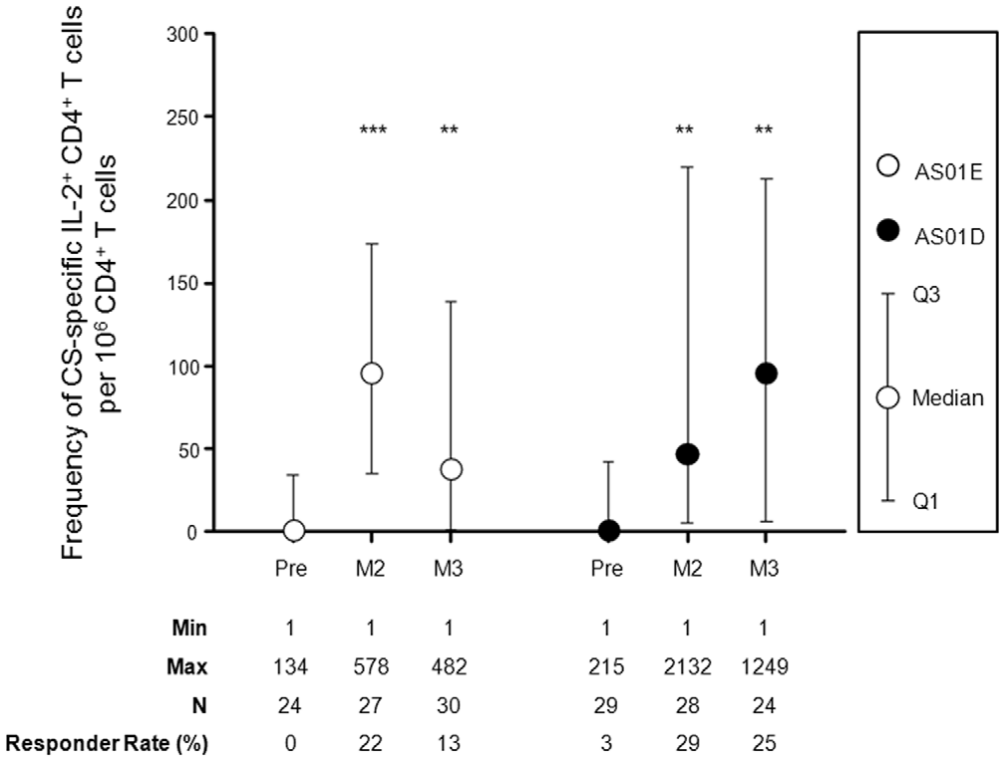
Induction of CD4^**+**^ T cell responses following vaccination with AS01 and AS02 based RTS,S formulations. Frequency of CD4^+^ T cells expressing at least IL-2 were measured before vaccination, one month after the second vaccination (month 2), and one month (month 3) after the third vaccination (month 14). ICS data are represented for RTS,S/AS01_E_ and RTS,S AS02_D_ vaccine formulations, indicating Q1, Median, Q3. Significant differences against pre-values are indicated as *** p<0.001, ** p<0.01 and * p<0.05. Minimum and maximum frequencies of IL-2^+^ CD4^+^ T cell responses, the number of evaluated subjects and the responder rates are indicated.

Low frequencies of CS- specific CD4^+^ T cells expressing IL-2 were detected upon *in vitro* stimulation of whole blood in children vaccinated with RTS,S/AS01_E_ or RTS,S/AS02_D_ (Figure 4). In both study groups, the frequency of CS-specific IL-2^+^ CD4^+^ T cells was significantly higher at one month post dose 2 and one month post dose 3 compared to pre-vaccination values. No significant differences were detected between the CS-specific IL-2^+^ CD4^+^ T cell responses when comparing RTS,S/AS01_E_ and RTS,S/AS02_D_ vaccination, at any timepoint. Frequencies of CD4^+^ T cells expressing at least IFN-γ, TNF-α or CD40L were not significantly higher than baseline values (data not shown). No CS-specific CD8^+^ T cell responses were detected one month after vaccination (data not shown). Considering the low level of response seen at peak (one month post dose 3) for IL-2^+^ CD4^+^ T cells, ICS analysis at 12 months post dose 3 was not performed. For information, in children who had T cell responses assessed, the induction of anti-CS antibodies (data not shown) showed a similar pattern to what has been described for children in the B cell subset.

## Discussion

This study investigated the adaptive B and T cell immune responses induced by the RTS,S antigen formulated with two different adjuvant systems in 18 month to 4 year old children living in a malaria endemic region. This is the first study reporting significant amounts of *P. falciparum* CS-specific IgG^+^ memory B cells and IL-2^+^ CD4^+^ T cells detected upon short-term *in vitro* restimulation, after vaccination of young children with AS01_E_ and AS02_D_ based formulations of the RTS,S pre-erythrocytic vaccine candidate. Previous work has shown that in infants receiving RTS,S/AS02_D_, a significant IL-2 production was detected in the supernatant of blood lymphocytes upon long-term *in vitro* restimulation with a CS-derived peptide pool [30].

This study demonstrates the induction one month after doses 2 and 3 as well as persistence at month 12 after last immunization of CS- specific circulating memory B cell and CS-specific antibodies in RTS,S/AS01_E_ and RTS,S/AS02_D_ vaccinated children. Similar to the association between the levels of antibodies to serogroup C meningococcal polysaccharide and the levels of circulating memory B cells in infants vaccinated with the MenC glycoconjugate vaccine [29], these data suggest the replenishment of the plasma cell pool on a regular basis through differentiation of CS specific memory B cells [31]. An alternative model proposes that long lived plasma cells would be solely responsible for the maintenance of serum antibodies titers [32]–[34]. However, the short half-life (between 40 to 100 days) of long-lived plasma cells described in several animal and human studies [35]–[37] make a scenario in which long-lived plasma cells sustain antibody production without continuous replenishment from memory B cells unlikely.

After two vaccine administrations, a higher frequency of CS-specific memory B cells was induced with the RTS,S/AS01_E_ than with RTS,S/AS02_D_. This may suggest a stronger priming and induction of large size germinal centers by RTS,S/AS01_E_ as we showed also a trend towards higher anti-CS antibody titers in the recipients of RTS,S/AS01_E_ in the same trial [22]. Strong stimulation of germinal centers by primary course vaccination has been suggested as a relevant factor for the persistence of the Ag-specific antibodies and Ag-specific memory B cells. This is supported by a trend toward a sustainable protective level of antibodies to serogroup C meningococcal polysaccharide in children vaccinated with MenC vaccine one year after the the primary course in the 2/3 of children that elicited memory B cells after third vaccination [38]. The majority of study participants in both the RTS,S/AS01_E_ and RTS,S/AS02_D_ groups had a significant level of memory B cells after the second vaccine dose and maintained a significant level of CS-specific antibodies one year after the last vaccination. The higher frequency of CS-specific memory B cells induced with the RTS,S/AS01_E_ after the second dose may be associated with the higher immunogenicity and efficacy of this vaccine observed in studies conducted in animals and naïve adults [3], [10], [11].

In contrast to antibody responses [22], an effect of prior exposure to the hepatitis B vaccine on the memory B cell response to the CS antigen was not detected here. This should be interpreted with caution in view of the low numbers involved in this analysis. Additional investigations on the mechanisms underlying the observed influence of previous hepatitis B vaccination on the CS antibody level may be needed to discriminate whether previous hepatitis B vaccination affects short CS antibodies producing plasma cells.

The rationale for investigating CD4^+^ T cell responses in children is based on the observation that CS-specific CD4^+^ T cells are associated with increased protection in adults [3], [6]. When investigating the CD4^+^ T cell responses to the CS antigen in children vaccinated with RTS,S/AS01_E_ or RTS,S/AS02_D_, using a whole blood assay, IL-2 expression by CD4^+^ T cells was the only significant response detected. CD40L expression was not observed. While the children in this study had detectable CS-specific IL-2^+^ CD4^+^ T cells, these responses were lower than those seen, using a PBMC based assay, in malaria naïve adults vaccinated with RTS,S/AS01 or RTS,S/AS02 [3]. Reduced expression of cytokines such as IFN-γ and TNF-α have been documented following vaccination of infants and are thought to be related to the somewhat immature status of the immune system [39]–[41]. Technical hurdles associated with CMI assessment in the field cannot be ruled out. CS-specific CD8^+^ T cells were not detected at one month post dose 2 and 3, in agreement with the absence of a detectable CD8^+^ T cell response to vaccination with RTS,S/AS01 or RTS,S/AS02 in adults [3].

Production of IL-2 has been linked to the establishment of memory T cell responses. It has been suggested that IL-2 and IFN-γ producing T cells after vaccination of adults are associated with the induction of memory T cell responses to pre-erythrocytic malaria antigens [42], [43]. In a recent RTS,S vaccine challenge trial, Kester and colleagues demonstrated that the frequency of CS-specific CD4^+^ T cells producing at least 2 immune markers amongst IL-2, TNF-α, IFN-γ and CD40L was significantly higher in protected subjects as compared to non-protected subjects [3].

Helper T cells promote antibody class switch [44], [45], affinity maturation [46] and induction of memory B cells [47] which play an important role in the induction of high titers of antigen specific IgG upon secondary exposure. In previous work, a fourth vaccination of Gambian adults one year after last vaccination with RTS,S/AS02 could significantly boost the anti-CS antibody response, suggesting that CS-specific memory had been induced [27].

IL-2 produced by antigen-specific CD4^+^ T cells may act as a growth factor for follicular helper T cells [48] and promote the growth and differentiation of antigen-specific B cells [49]. Follicular helper T cells (T_FH_) are now recognized as the subset of helper T cells that regulate the multiple stages of the B cell compartment [50]. Upon contact with antigen-primed dendritic cells, T_FH_ migrate to the follicular regions of lymphoid organs to engage in cell-to-cell contacts with antigen-primed B cells. CD40L, a marker for activated antigen-specific CD4^+^ T cells is a co-stimulatory molecule which is vital in the delivery of T_FH_ cell help for the development of antigen-specific memory B cells [51]. It is unclear why CD40L upregulation was not detected upon antigen activation. Other experimental approaches may be necessary. Despite undetectable CD40L expression on circulating CS specific CD4^+^ T cells observed in this study, we found that all children had detectable and persistent levels of anti-CS serum IgG after vaccination as well as CS-specific memory B cells up to one year after last vaccination.

Together, these data show that vaccination with RTS,S/AS01_E_ and RTS,S/AS02_D_ induces significant CS-specific long term humoral immunity as well as CS-specific IL-2 producing CD4^+^ T cells in young children. While CD40L was undetectable, the induction and persistence of CS-specific memory B cells in all recipient suggest that both RTS,S/AS01_E_ and RTS,S/AS02_D_ vaccines engaged CD4^+^ T helper (T_FH_) in the development of CS-specific B cells. These mechanisms may occur with a greater amplitude following the vaccination with RTS,S/AS01_E_ as it generated higher frequency of CS-specific memory B cells and higher level of CS-specific antibodies than RTS,S/AS02_D_ and could explain the finding that the AS01_E_ adjuvant improved the RTS,S vaccine efficacy as shown in the sporozoite challenge model [3]. In addition, CS-specific IL-2^+^ CD4^+^ T cells are linked to the memory T cell compartment and may be involved in RTS,S/AS01_E_ and RTS,S/AS02_D_ vaccine-induced long term protection. Further immunological investigations in RTS,S studies, including efficacy studies, will contribute to a better understanding of vaccine-induced protection against malaria.

## Supporting Information

Checklist S1 CONSORT Checklist.
(DOC)
